# D-Dimer levels are correlated with disease activity in Crohn's patients

**DOI:** 10.18632/oncotarget.19250

**Published:** 2017-07-12

**Authors:** Junwu Zhang, Zhen Guo, Wei Yang, Zhongliang Zhu, Wanzhong Kong, Sujie Zheng, Lei Jiang, Xianming Fei, Yanxia Chen, Jinlin Liu

**Affiliations:** ^1^ Department of Clinical Laboratory, Wenzhou Traditional Chinese Medicine Hospital, 325000 Wenzhou, China; ^2^ Department of Clinical Laboratory, Second Affiliated Hospital, School of Medicine, Zhejiang University, 310009 Hangzhou, China; ^3^ Department of Clinical Laboratory, Zhejiang Provincial People's Hospital, Hangzhou Medical College, 310014 Hangzhou, China; ^4^ Department of Rheumatology, Zhejiang Provincial People's Hospital, Hangzhou Medical College, 310014 Hangzhou, China; ^5^ Key Laboratory of Tumor Molecular Diagnosis and Individualized Medicine of Zhejiang Province, 310014 Hangzhou, China

**Keywords:** plasma d-dimer, coagulation function, fibrinolysis, Crohn’s disease, CDAI

## Abstract

Various indices have been used to assess Crohn's disease (CD). However, the question of whether the Crohn's Disease Activity Index (CDAI) is associated with coagulation function has not been fully confirmed. In this study, we examined the association between CDAI and the coagulation and fibrinolysis parameters. In a retrospective and observational cohort study, the CDAI of 108 patients from two hospital centers was calculated, and its correlations with the prothrombin time (PT), activated partial thromboplastin time (APTT), thrombin time (TT), international normalization ratio (INR), fibrinogen (Fg) and plasma D-Dimer were investigated. Significant differences were found for PT, APTT, TT, INR, Fg and D-Dimer between the healthy controls and CD patients. However, no significant difference was found between the CDAI-High and CDAI-Low groups of CD patients. Moreover, the CDAI was positively correlated with the level of D-Dimer in CD patients of two hospitals, regardless of the detection method (hospital 1: r=0.3268, *p*= 0.0042; hospital 2: r=0.5553, *p*=0.0008). Among the blood coagulation and fibrinolysis parameters, the D-Dimer level was highly correlated with CDAI in CD patients. Thus, the level of D-Dimer expression may be a promising new marker for assessing CD disease activity.

## INTRODUCTION

Inflammatory bowel disease (IBD) is a chronic, relapsing, and inflammatory disorder of the gastrointestinal tract that includes two conditions: Crohn's disease (CD) and ulcerative colitis (UC). Currently, it is well recognized that CD is associated with an increased risk for the development of a number of extra-intestinal manifestations, which include skin, joint and eye manifestations [1, 2]. Of the extra-intestinal manifestations of CD, thromboembolics are a major cause of morbidity and have a 3.6 times increased risk for occurrence compared to the general population [2, 3]. Generally, acquired endothelial dysfunction, coagulation system activation, impaired fibrinolysis and platelet abnormalities are the main characteristics of the hypercoagulable status of CD patients [4]. Moreover, this hypercoagulable state is followed by an increased risk of venous thromboembolism (VTE) which is also associated with the disease activity [4]. Therefore, thromboprophylaxis is strongly recommended for the clinical management of CD patients, especially hospitalized patients [4, 5].

An abnormal activation of coagulation and fibrinolysis is often encountered among CD patients, and such patients are associated with worse outcomes [6]. Studies of the hypercoagulable status in IBD have provided varying results. These studies have revealed that almost all components of hemostasis, such as fibrinolysis, platelets and the coagulation cascade, participate in its pathogenesis [4, 7]. Obviously, more research is needed to elucidate these complex interactions and determine the clinical significance of these coagulation parameters in CD patients [8]. Therefore, the aim of this study is to explore the potential clinical value of blood coagulation and fibrinolysis parameters for CD patients. In this study, 108 CD patients and 108 healthy controls from two hospitals were recruited, and various coagulation and fibrinolysis laboratory parameters and their correlations with the Crohn's disease severity (CDAI) were investigated, including prothrombin time (PT), activated partial thromboplastin time (APTT), thrombin time (TT), international normalization ratio (INR), fibrinogen (Fg) and the plasma D-Dimer.

## RESULTS

### Patients with CD versus controls

To avoid exclude differences in coagulation and fibrinolysis results due to different detection reagents and systems, we enrolled 108 CD patients from two different hospitals (hospital 1: The Second Affiliated Hospital of Zhejiang University School of Medicine and hospital 2: Zhejiang Provincial People's Hospital of Hangzhou Medical College), where the detection system of hospital 1 was a Stago STA-R system. The detection system of hospital 2 was a Symex 5100, and the reagents were from the SIEMENS company. The demographic and clinical characteristics of the inpatient and ambulatory patients are shown in Table 1, and 108 age-matched healthy controls were investigated. The median (IQR) age of CD patients was 34 years (26-43) old, and nearly half of the patients were men (41%). The proportion of patients at hospital 1 and hospital 2 was 69.4% and 30.6%, respectively. The median (IQR) of the CDAI scores of the two hospitals with CD patients was 124.1 (79.0-175.2) and 162.0 (102.5-200.0), respectively (Table 1).

**Table 1:** Baseline characteristics of the total study population

	Healthy controls	CD patients
No. of subjects	108	108
Age,years		
Median (range)	48(44-52)	34(26-43)
≤60	97%	94%
>60	3%	6%
Gender		
Male	49	64
Female	59	44
Hospital (No. of subjects)		
Hospital 1	65	75
Hospital 2	43	33
CDAI		
Hospital 1	ns	124.1(79.0-175.2)
Hospital 2	ns	162.0(102.5-200.0)
Disease duration	ns	2.0 (1.0-6.0)
Location of disease		
Small intestine	ns	54(50.0%)
Colon	ns	47(43.5%)
Rectum	ns	7(6.5%)
Anal fistula	ns	0(0%)
Medication within one month before the blood draw		
No medication	ns	75(69.4%)
5-ASA	ns	20 (18.5%)
Corticosteroids	ns	3(2.8%)
Immunosuppressor	ns	12.0(11.1%)
TNF-α inhibitor	ns	3(2.8%)

**ns:** no significant.

As therapy regimens overlapped, the total is 104.6 %.

### Significant difference of coagulation laboratory markers between CD patients and healthy controls

Table 2 shows that the PT, APTT, INR, Fg, and plasma D-Dimer of patients with CD were significantly elevated compared to those of healthy controls (all *p*<0.0001). However, the thrombin time (TT) levels of CD patients were significantly decreased compared to the healthy controls (*p*<0.0001). Together, these abnormal coagulation and fibrinolysis parameters revealed the hypercoagulable status of CD patients.

**Table 2:** Comparisons of coagulation and fibrinolysis parameters between CD patients and controls

Coagulation tests	CD Patients (n=108)		Controls (n=108)		*P*
	Median	IQR	Median	IQR	
D-dimer (ug/L)	335.0	235.0-457.5	140.0	92.25-227.5	<0.0001
Fibrinogen (mg/dl)	3.540	2.855-4.220	2.515	2.193-2.810	<0.0001
PT (sec)	13.10	12.40-13.80	11.30	10.73-11.90	<0.0001
APTT (sec)	37.70	33.35-41.90	27.40	25.40-30.20	<0.0001
TT (sec)	16.00	15.13-17.20	18.70	17.80-19.78	<0.0001
INR	1.055	1.000-1.118	0.980	0.930-1.01	<0.0001

**Reference values:** D-dimer (ug/L): 0-550. PT: prothrombin time (sec): 9.99-13.52. APTT: activated partial thromboplastin time (sec): 20.5-30.8. TT: thrombin time (sec): 13.9-20.9. INR: international normalization ratio: 0.85-1.15. Fg: fibrinogen (mg/dl): 2.0-4.0. Results are presented as median (IQR).

### No difference in coagulation laboratory markers between CDAI-H and CDAI-L patients

CD patients were assigned to either the high (CDAI-H, CDAI≥150) or low (CDAI-L, CDAI<150) disease activity group (Table 3). All coagulation and fibrinolysis laboratory markers were investigated in each group. However, there were no differences in these abnormally elevated values of coagulation and fibrinolysis markers between active and inactive CD patient status with PT (*p*=0.708), TT (*p*=0.867), APTT (*p*=0.204), INR (*p*=0.602), Fg (*p*=0.119) and plasma D-Dimer (*p*=0.11) (Table 3).

**Table 3:** Comparisons of coagulation and fibrinolysis parameters between CDAI-H and CDAI-L CD Patients

Coagulation tests	CDAI≥150 (n=47)		CDAI<150 (n=61)		*P*
	Median	IQR	Median	IQR	
D-dimer (ug/L)	380.0	290.0-700.0	280.0	225.0-420.0	0.110
Fibrinogen (mg/dl)	3.670	2.960-4.480	3.430	2.695-3.975	0.119
PT (sec)	13.1	12.2-13.9	13.1	12.6-13.7	0.708
APTT (sec)	36.60	31.40-41.90	39.10	34.75-42.05	0.204
TT (sec)	15.90	15.10-17.30	16.00	15.25-16.80	0.867
INR	1.070	1.000-1.120	1.040	1.000-1.115	0.602

**Reference values:** D-dimer (ug/L): 0-550. PT: prothrombin time (sec): 9.99-13.52. APTT: activated partial thromboplastin time (sec): 20.5-30.8. TT: thrombin time (sec): 13.9-20.9. INR: international normalization ratio: 0.85-1.15. Fg: fibrinogen (mg/dl): 2.0-4.0. Results are presented as median (IQR).

### Plasma D-Dimer was significantly correlated with the CDAI of CD patients

The above results indicate that CD patients had abnormal coagulation and fibrinolysis status. We then wondered whether these abnormal coagulation parameters correlated with the CDAI scores in these CD patients. First, we pooled together these patient coagulation and fibrinolysis parameters for the two hospitals and analyzed the correlation coefficient with the CDAI of these 108 CD patients (Table 4). Herein, our results show that there was no difference for the PT (r =-0.0164, *p* =0.8662), TT (r =-0.0156, *p* =0.8727), APTT (r =-0.1146, *p* =0.2374), INR (r =0.0831, *p* =0.3923), or Fg (r =0.1665, *p *=0.0851) but that the levels of plasma D-Dimer are significantly correlated with the CDAI (r =0.3562, *p* =0.0002) (Table 4). Next, we analyzed the correlation coefficient with the CDAI in the two hospitals separately. These results showed that the levels of plasma D-Dimer were significantly correlated with the CDAI (hospital 1: r =0.3268, *p* =0.0042; hospital 2: r =0.5553, *p* =0.0008). Additionally, no significant difference was found for the PT, TT, APTT, INR or Fg in the two hospitals separately (Table 4). Overall, our results clearly show that the levels of plasma D-Dimer were significantly correlated with the CDAI, despite the use of different reagents and detection systems in the two hospitals.

**Table 4:** Correlation coefficient between the coagulation and fibrinolysis parameters with the CDAI of the CD patients

Value	Hospital 1 (n=75)		Hospital 2 (n=33)		Total (n=108)	
	r	*p*	r	*p*	r	*p*
D-dimer	0.3268	0.0042	0.5553	0.0008	0.3562	0.0002
Fibrinogen	0.2212	0.0566	−0.0567	0.7540	0.1665	0.0851
PT	0.1787	0.1250	−0.1712	0.3409	−0.0164	0.8662
APTT	0.0554	0.6368	−0.1595	0.3754	−0.1146	0.2374
TT	−0.1740	0.1354	−0.2485	0.1632	−0.0156	0.8727
INR	0.1835	0.1151	−0.2673	0.1326	0.0831	0.3923

## DISCUSSION

The assessment of CD activity is based on a combination of clinical symptoms and endoscopy. Several laboratory markers have been evaluated for monitoring CD patient's activity. Active inflammation in CD patients is associated with an acute-phase reaction. The non-specific inflammation marker, C-reactive protein (CRP), which is produced by hepatocytes upon stimulation by proinflammatory cytokines, has been found to be associated with clinical and endoscopic activity in IBD. CRP seemed to be more sensitive than CDAI in the evaluation of patients with CD [10, 11]. Furthermore, it has long been known that acute inflammation, as a response to infection or trauma, can lead to activation of the coagulation system. Thus, the results of this study demonstrate that the non-specific fibrinolysis factor D-Dimer may be another disease activity parameter for CD patients.

Patients with CD exhibit a higher risk of developing systemic thrombosis than the general population, and this risk is associated with a hypercoagulable status and VTE [4, 6, 12]. In particular, the hypercoagulable state has been associated with active IBD [2, 13]. However, in other studies, the activation of coagulation was observed in both active and inactive IBD [4, 14]. To our knowledge, for the hypercoagulable states, the PT and APTT values of CD patients should be shorter than those of the healthy control. However, *in vitro* laboratory tests may not predict the actual condition of CD patients *in vivo*. Consistent with previous studies, we confirm that coagulation parameters, including the PT, TT, APTT, INR and Fg, were all significantly elevated compared with healthy controls.

Moreover, activation of the coagulation system results in fibrin formation, which is followed by lysis and breakage of the fibrin clot to fibrin degradation products [15]. This fibrinolytic system is mainly regulated by a balance between tissue-type plasminogen activator and plasminogen activator inhibitor-1 [15]. In IBD patients, the fibrinolysis system has been found to play an important role in hypercoagulability [16]. Additionally, several groups have shown that hypofibrinolysis is common to both UC and CD patients and may be potentially implicated in the observed hypercoagulable state of these diseases [4, 17].

D-dimer is a fibrin-derived fragment that is released into the blood when cross-linked fibrin is broken down by the fibrinolytic system [18]. And the D-dimer assay has gained significance as a tool that assists in clinical decisions on the presence of thrombosis [19]. Increased D-dimer levels are associated with a tendency towards thrombus, which increases the risk of pulmonary embolism, deep vein thrombosis or disseminated intravascular coagulation [15]. For IBD diseases, many articles have been published with intensive discussion on the topic. However, studies on hypercoagulable status in a small cohort of patients have provided varying results. Biancone L [20] investigated the D-Dimer level of only 24 CD patients and 14 healthy subjects. A study revealed increased D-dimer levels in 24 active UC patients when compared to 18 patients with inactive disease [21]. Similarly, another study reported higher levels of D-dimer detection in 12 CD patients when compared to 20 healthy controls [22]. However, Nguyen [23] uncovered a high prevalence of elevated D-dimer in deep venous thrombosis (DVT)-negative IBD patients, yet these results limit its utility of this group of patients. Dolapcioglu [8] examined the coagulation parameters of IBD patients, but only 18 CD patients and 26 healthy controls were included in this study. In one study [24], 123 CD patients were included, but only 36 CD patients had undergone the D-dimer test; this study focused primarily on the effects of infliximab therapy on coagulation functions. Additionally, the prothrombotic clot phenotype might represent a novel mechanism that increases thrombotic risk in IBD [25]. Moreover, the authors did not analyze D-Dimer or its correlation with disease activity of the CD patients. Importantly, only one study [17] showed that D-dimers were significantly correlated with disease activity of the UC with active status. Above all, the number of IBD patients in the above studies was relatively small, and the studies often pooled together patients with UC and CD disease and did not separate the different conditions.

Conversely, our study had 108 patients in two different hospital centers that utilized different detection systems. The results show that plasma D-Dimer of CD patients was significantly elevated compared with the healthy controls. No significant difference was observed between CD patients with active and inactive disease status based on the Mann-Whitney U test, which was used to compare whether two sample means are equal or not. However, the Pearson's rank correlation showed a linear relationship between the sets of data. Hence, further analysis clearly showed that the levels of plasma D-Dimer were significantly correlated with the CDAI, regardless of the use of different reagents and detection systems in the two hospitals.

Overall, in the present study, six coagulation parameters encompassing the coagulation and fibrinolysis indexes involved in thrombus formation were assessed. We found that only the levels of D-dimer were significantly correlated with CDAI, which highlights it as a potential disease marker for assessing the CDAI of CD patients.

## MATERIALS AND METHODS

### Patients

In total, 108 patients with CD (49 males, 59 females) and 108 healthy controls (64 males, 44 females) from two hospitals (hospital 1: The Second Affiliated Hospital of Zhejiang University School of Medicine and hospital 2: Zhejiang Provincial People's Hospital of Hangzhou Medical College) were included in this study (Table 1). The study was approved by the Zhejiang Provincial People's Hospital and The Second Affiliated Hospital of Zhejiang University School of Medicine Review Board. Patients gave informed consent for their sample analysis, in accordance with the Declaration of Helsinki. The diagnosis of CD was based on clinical, endoscopic, histological, radiological and microbiological criteria. Exclusion criteria for all participants were (a) bacterial or viral infection; (b) thromboembolism within the last three months; and (c) continuous anticoagulation with low-molecular-weight heparins or vitamin K antagonists. For patients with progression, additional exclusion criteria were (d) surgery or radiotherapy within 2 weeks and (e) severe renal or hepatic insufficiency, valvular disease, atrial fibrillation, pregnancy and oral contraceptive prior to study [8].

### CDAI assessments

Crohn's disease severity was evaluated using CDAI [9], where a score <150 was considered inactive and a score ≥150 was considered active. In addition to medical history and a thorough physical examination, all CD patients underwent chest X-ray, lower extremity or abdominal Doppler USG examinations to identify any evidence of thromboembolism. Then, the CD patients were assigned to high (CDAI-H, CDAI≥150) or low (CDAI-L, CDAI<150) disease activity groups.

### Clinical parameters

Venous blood samples were collected from all study participants, placed in tubes containing 3.8% trisodium citrate, and centrifuged at 2000 rpm for 10 minutes; we then separated the plasma. The plasma levels of PT, APTT, TT, Fg, INR and D-dimer in Hospital 1 were measured using a Stago STA-R machine, and the reagent was from the Stago company. The levels in Hospital 2 was measured using the Symex 5100 system, and reagents were purchased from SIEMENS company. Additionally, monoclonal antibody detection of the turbidimetric inhibition immunoassay method was used for D-Dimer detection.

### Statistical analysis

Statistical analysis was performed using GraphPad Prism software and the following statistical tests were performed: the Mann-Whitney U test and Pearson's rank correlation. In addition, quantitative data are presented as the median (IQR).

## Abbreviations

IBD: inflammatory bowel disease
UC: ulcerative colitis
CD: Crohn's disease
VTE: venous thromboembolism
CDAI: Crohn's Disease activity index
PT: prothrombin time
APTT: activated partial thromboplastin time
TT: thrombin time
INR: International normalization ratio
Fg: fibrinogen
